# Person-First or Disease-First? Language Choices in Cancer Communication

**DOI:** 10.3390/nursrep16040143

**Published:** 2026-04-16

**Authors:** Anna Tsiakiri, Konstantinos Tzanas, Despoina Chrisostomidou, Spyridon Plakias, Foteini Christidi, Christos Frantzidis, Nikolaos Aggelousis, Maria Lavdaniti, Evangeli Bista

**Affiliations:** 1 Department of Neurology, School of Medicine, Democritus University of Thrace, 68100 Alexandroupolis, Greece; 2 Cancer Patient Guidance Center (KAPA3), 11141 Athens, Greece; ktzanas96@gmail.com (K.T.); education@kapa3.gr (D.C.); libista@yahoo.gr (E.B.); 3 Department of Physical Education and Sport Science, University of Thessaly, 41500 Trikala, Greece; splakias@uth.gr; 4 Department of Psychology, School of Philosophy, National and Kapodistrian University of Athens, 15784 Athens, Greece; fchristidi@psych.uoa.gr; 5 School of Engineering and Physical Sciences, University of Lincoln, Brayford Pool, Lincoln LN6 7TS, UK; cfrantzidis@lincoln.ac.uk; 6 Department of Physical Education and Sport Science, Democritus University of Thrace, 69100 Komotini, Greece; nagelous@phyed.duth.gr; 7 Nursing Department, International Hellenic University, Sindos, 57400 Thessaloniki, Greece; maria_lavdaniti@yahoo.gr

**Keywords:** cancer communication, illness identity, stigma, person-first language, psychosocial oncology, health terminology, mixed-methods research, narrative medicine, patient experience, medical discourse

## Abstract

**Background/Objectives**: Cancer-related terminology is not merely descriptive and plays a critical role in shaping emotional responses, personal identity, and communication across clinical, social, and public spheres. Despite growing interest in the psychosocial dimensions of illness language, few studies have centered the lived experiences of individuals navigating cancer through the lens of terminology. This study explores how people living with and beyond cancer perceive, interpret, and emotionally respond to cancer-related language, focusing on the way terminology influences identity, stigma, and communicative interaction. **Methods:** A sequential mixed-methods design was employed. The quantitative phase involved 146 participants with a cancer diagnosis completing a structured questionnaire on preferred terminology and emotional impact. The qualitative phase followed, using open-ended questionnaires with 11 participants to deepen understanding of linguistic experiences. Thematic content analysis was used to identify patterns across narratives. **Results:** These findings reveal that labels such as “cancer patient” evoke strong negative emotional reactions, associated with stigma, fear, and identity reduction. Person-first and context-sensitive language was perceived as more respectful and empowering. Emotional responses to language varied widely, from fear to neutrality, shaped by speaker role, context, and time since diagnosis. Media representations were often seen as dramatizing or moralizing, reinforcing the need for communicative clarity, empathy, and education in both clinical and public discourse. **Conclusions:** Cancer-related language is a powerful psychosocial force. It shapes how individuals are seen and see themselves and can either reinforce stigma or foster dignity and resilience. This study highlights the urgent need for person-centered, context-aware communication practices across healthcare, media, and society.

## 1. Introduction

Cancer represents a critical public health burden, yet accumulating evidence demonstrates that its impact extends far beyond biomedical pathology, constituting a complex social and psychosocial experience shaped by fear, stigma, uncertainty, and relational dynamics. Research on cancer screening and early diagnosis highlights how emotional responses, social beliefs, and communication practices influence health-seeking behaviours, often acting as barriers or facilitators to timely care [1,2]. Across the illness trajectory, from diagnosis through treatment and into survivorship or long-term management, patients navigate fluctuating emotional states, evolving identities, and changing support needs, underscoring cancer as a temporally dynamic experience rather than a discrete medical event [3,4]. Emerging evidence from survivorship and digital care contexts further illustrates how trust, reassurance, and perceived empathy within communicative interactions shape adaptation and continuity of care [5]. These findings support a shift from viewing cancer solely as disease to understanding it as an illness experience, embedded within social relations and mediated through communication across all phases of the cancer journey.

In healthcare contexts, language operates as an active constitutive force that shapes meaning, expectations, and emotional responses, rather than as a neutral medium for conveying biomedical facts. Qualitative and mixed-methods research demonstrates that the ways illnesses are named, explained, and narrated influence how patients understand risk, interpret symptoms, and position themselves within the healthcare system [1,6,7]. Medical terminology functions as a powerful framing mechanism, capable of normalizing uncertainty and fostering trust or, conversely, intensifying fear, stigma, and disengagement, particularly in culturally sensitive or stigmatized conditions [8,9]. Studies of cancer communication further show that labels and standardized clinical language can shape expectations about prognosis, responsibility, and survivorship, thereby eliciting distinct emotional reactions such as anxiety, shame, or reassurance [10,11]. These dynamics connect directly with theoretical concepts of stigma and labeling, which emphasize how diagnostic categories acquire social and moral meanings, as well as with narrative medicine, which foregrounds storytelling and interpretive agency as central to illness experience.

Cancer-related terminology constitutes a central mechanism through which identity is constructed, negotiated, and contested across the cancer trajectory. Empirical research demonstrates that commonly used labels such as “cancer patient” and “cancer survivor” carry distinct semantic and moral implications, shaping how individuals understand themselves and how they are positioned socially and clinically [12]. While disease-centered labels may provide clinical clarity, they can also reduce the individual to the diagnosis, foregrounding illness as a dominant identity marker and constraining narrative agency, particularly in chronic or metastatic contexts [13]. Qualitative studies of survivorship further show that identity is not static but evolves through embodied and narrative processes, as individuals move between illness-focused, coexistence, and integrative self-understandings [14]. In response to the identity-limiting effects of diagnostic labeling, person-first language has been proposed as an ethical and communicative alternative that decouples personhood from pathology and allows greater narrative flexibility. At the same time, critical analyses of cancer discourse highlight the risks of heroization and victimization, showing how dominant metaphors of battle, survival, or suffering impose moral expectations and obscure ambivalence, vulnerability, and diversity of experience [15].

Cancer-related language carries substantial psychological weight, functioning as an emotional trigger that can evoke fear, fatalism, empowerment, or neutrality depending on context and individual positioning. Empirical research shows that certain words and phrases associated with cancer are commonly linked to anxiety, threat, and anticipated suffering, particularly when embedded in stigmatizing or crisis-oriented discourses [16]. For example, expressions such as “terminal illness,” “malignant disease,” or metaphors of “battle” and “fighting cancer” may evoke fear, fatalism, or a sense of impending loss, especially when they imply inevitability, personal responsibility, or limited control over outcomes. Similarly, labels such as “cancer victim” may reinforce passivity and vulnerability, contributing to stigma and emotional distress. At the same time, studies within psycho-oncology demonstrate that language can also serve as a resource for empowerment, supporting coping, meaning-making, and emotional regulation when framed in ways that acknowledge uncertainty while preserving agency [17,18]. For instance, person-centered expressions such as “person living with cancer,” “undergoing treatment,” or supportive phrases such as “I am here with you” or “we will manage this together” have been associated with feelings of validation, relational support, and greater perceived control. Such linguistic choices may facilitate adaptive coping, reduce emotional burden, and support the integration of illness into a broader sense of self. Importantly, the emotional impact of cancer language is not uniform: it varies according to stage of disease, time elapsed since diagnosis, and individual coping mechanisms, with neutral or attenuated emotional responses more frequently reported among individuals with longer illness trajectories or greater narrative integration [19]. These findings underscore that words do not carry fixed emotional valence; rather, their effects are shaped by experiential, temporal, and relational factors.

The impact of cancer-related language is profoundly shaped by who speaks and within which communicative context. Health professionals occupy a position of epistemic and moral authority, and their linguistic choices strongly influence patients’ trust, emotional security, and sense of being understood. Empirical studies demonstrate that clear, empathetic, and patient-centred communication by clinicians can buffer fear and hopelessness, whereas ambiguous or emotionally detached language may exacerbate distress and undermine trust [20,21]. Beyond the clinical setting, communication within the social environment often reflects good intentions but may simultaneously generate emotional burden, particularly when shaped by stigma, cultural taboos, or avoidance of open discussion [22]. At the level of public discourse, mass media and social media constitute powerful yet unstable communicative spaces, frequently characterised by stereotyping, dramatization, information overload, and misinformation. Media analyses show that simplified or emotive narratives can reinforce stigma, shape public perceptions of “who gets cancer,” and influence help-seeking behaviours, sometimes with unintended harmful effects [23].

Despite growing recognition of language as a psychosocial determinant in cancer care, existing research remains fragmented, with limited integration of quantitative and qualitative approaches capable of capturing both patterned responses and lived meaning. Most studies examine communication outcomes (e.g., satisfaction, trust, distress) without systematically analysing the linguistic content itself, while qualitative work often foregrounds narratives without linking them to measurable emotional or attitudinal effects. Moreover, research rarely centres the perspectives of people living with cancer as primary interpreters of cancer-related language, instead privileging clinician-defined or media-driven frameworks [20,21]. A further critical gap concerns the scarcity of evidence from specific linguistic and cultural contexts, where cancer terminology carries locally embedded meanings, moral connotations, and stigma, contexts that remain underrepresented in international literature [22,24]. In particular, data from Greek-language and Greek cultural settings are virtually absent, limiting the transferability of existing communication models. Addressing these gaps, the present study adopts a mixed-methods design grounded in participants’ own linguistic experiences, with the aim of generating empirically informed communication recommendations that are culturally sensitive, clinically relevant, and directly applicable to cancer care practice and public discourse.

The aim of the present study is to systematically examine the experiential, emotional, and identity-related impact of cancer-related language, as articulated by people living with and beyond cancer. Moving beyond purely outcome-focused approaches, the study seeks to foreground participants’ own interpretations of cancer terminology and to explore how language operates as a psychosocial force within everyday communication. Specifically, the study addresses the following research questions: (1) How is cancer-related terminology experienced and interpreted by individuals with cancer? (2) In what ways does such terminology shape identity, emotional responses, and communicative interactions across clinical, social, and public contexts? and (3) What linguistic preferences do participants express regarding labels, descriptors, and communicative framing? To address these questions, the study adopts a mixed-methods design, integrating quantitative patterns with qualitative depth, thereby enabling a nuanced analysis that captures both the distribution and the lived meaning of language-related experiences.

## 2. Materials and Methods

### 2.1. Study Design

The research was conducted with beneficiaries of the Cancer Patient Guidance Center (KAPA3) (http://www.kapa3.gr, accessed on 3 December 2025), a Greece-based non-profit organization that provides support, patient navigation services, education, and advocacy for individuals living with cancer and their families. KAPA3 primarily operates within the Greek healthcare and social context, while also engaging in broader outreach through digital platforms, awareness initiatives, and collaborations at national and international levels. The study was conducted as part of an effort to inform policy development without thematic or geographical exclusions, while maintaining high scientific standards. The study actively engaged citizens in a scientific endeavor that generates new knowledge and/or perspectives. In this context, KAPA3 collaborators, including trained staff members and patient advocates with lived experience of cancer, participated not only as contributors and partners, but also as active collaborators in the research process. Their involvement followed a participatory research approach and included contributions to the development and refinement of the study design and questionnaire, as well as to the interpretation and contextualization of findings.

To address potential ethical considerations related to dual roles, clear distinctions were maintained between collaborators and study participants. Participation in the study was voluntary and anonymous, and no individuals involved in the research design or implementation were included as participants in the analyzed dataset. All procedures were conducted in accordance with institutional ethical approval and principles of confidentiality, role transparency, and respect for participants’ autonomy. Particular attention was given to the management of dual roles within the participatory framework, ensuring that collaborators involved in the research process were not included as study participants and that no undue influence was exerted on recruitment or responses.

This study followed a sequential mixed-methods design, combining a quantitative survey with a qualitative content analysis to explore perceptions and experiences related to cancer-related language. The quantitative phase was conducted first and was subsequently used to inform the qualitative phase, allowing for in-depth exploration and contextualization of the survey findings. Specifically, a quantitative cross-sectional questionnaire was administered to a large sample of individuals with a cancer diagnosis, followed by a qualitative inquiry involving participants selected for deeper exploration of meanings, interpretations, and lived experiences related to language use.

### 2.2. Participants and Data Collection

#### 2.2.1. Quantitative Phase (Phase I)

The first phase of the study consisted of a non-probability convenience sample of 146 individuals diagnosed with a neoplastic disease and registered in the K3 Registry. Data collection took place between June and August 2024. Data were collected using a self-administered structured questionnaire comprising five closed-ended questions. The questionnaire focused on: preferred terminology used by third parties to describe one’s health condition, emotional impact of the terms “patient” and “cancer patient,” basic demographic variables (gender and year of birth). Participation was voluntary and anonymous. An introductory information note explained the purpose of the study, data protection measures, and the intended use of the findings for statistical reporting and communication recommendations. The questionnaire items were developed based on existing literature on cancer communication, illness identity, and psychosocial oncology, as well as the study’s conceptual framework and research questions. The content was refined through iterative discussion within the research team, including input from KAPA3 collaborators with lived experience, to ensure clarity, relevance, and contextual appropriateness. The final instrument consisted of five closed-ended questions focusing on terminology preferences and emotional responses. An outline of the questionnaire items is provided in Supplementary Materials (https://doi.org/10.5281/zenodo.19512148 accessed on 3 December 2025). Participants were recruited through the Cancer Patient Guidance Center (KAPA3) using its existing communication channels, including direct email invitations, website announcements, and social media outreach. Individuals registered with or engaged in KAPA3 services were invited to participate voluntarily. No financial or other incentives were provided. Participation was based on self-selection following receipt of study information.

#### 2.2.2. Statistical Analysis (Phase I)

Quantitative data were analyzed using IBM SPSS Statistics software (Version 29, IBM SPSS Inc., Chicago, IL, USA). Descriptive statistics were computed to summarize participants’ sociodemographic characteristics and responses to illness-related terminology. Frequencies and percentages were calculated for categorical variables, while means, standard deviations, and ranges were computed for continuous variables. Given the ordinal nature of the variables assessing emotional impact of terminology, non-parametric statistical tests were selected. Specifically, a Wilcoxon signed-rank test was used to compare emotional responses to the paired terms “patient” and “cancer patient,” allowing for within-participant comparison without assuming normality.

To examine factors associated with participants’ preferred illness-related description, a multinomial logistic regression analysis was conducted. Preferred terminology served as the dependent variable, with “indifferent” set as the reference category. Age and gender were included as sociodemographic predictors, while emotional impact ratings for the terms “patient” and “cancer patient” were entered as independent variables. Model fit was assessed using likelihood ratio tests, and effect estimates were reported using odds ratios with corresponding confidence intervals. Additional ordinal logistic regression analyses were performed to explore potential associations between sociodemographic variables (age and gender) and emotional responses to illness-related terminology. These analyses did not yield statistically significant results and are therefore reported descriptively.

All statistical tests were two-tailed, and statistical significance was set at *p* < 0.05.

#### 2.2.3. Qualitative Phase (Phase II)

Findings from the quantitative phase informed the design of the qualitative phase.

Participants in the qualitative phase were recruited from individuals who had already engaged with KAPA3 and expressed willingness to contribute further to the study. Recruitment involved an initial self-selection process, whereby individuals responded voluntarily to invitations disseminated through KAPA3 communication channels. From this pool, a purposive sampling approach was applied to ensure variation in demographic and clinical characteristics, including age, gender, cancer type, disease stage, and time since diagnosis. Invitations were communicated through KAPA3 channels, and participation was entirely voluntary and anonymous. All participants received information about the study aims and procedures prior to participation and provided informed consent. A total of 11 (P01–P11) participants were included in the final analysis. Demographic and clinical characteristics (e.g., age, education level, type of cancer, stage, time since diagnosis, and treatment history) were recorded separately to contextualize responses but were not subjected to qualitative coding.

The questionnaire consisted of open-ended questions addressing emotional responses to cancer-related terminology, perceptions of identity-related language, the role of the speaker and communicative context, the influence of media and public discourse and preferred terminology and recommendations for communication about cancer. The open-ended questions were designed to elicit in-depth reflections on language use, identity, emotional responses, and communication contexts, in alignment with the study’s research questions and relevant literature. The development process followed an iterative approach, incorporating both theoretical considerations and experiential input from KAPA3 collaborators. An outline of the qualitative questions is provided in Supplementary Materials (https://doi.org/10.5281/zenodo.19512148 accessed on 3 December 2025). All responses were anonymized and assigned unique participant identifiers (P01–P11).

#### 2.2.4. Analytical Approach for Phase II

A thematic content analysis was conducted following an iterative and systematic process. The unit of analysis was defined as a meaning unit, operationalized as a segment of text expressing a single coherent idea relevant to the research questions. A single response could therefore yield multiple meaning units. The analysis proceeded in four main stages:

1. Familiarization

All responses were read repeatedly to achieve immersion in the data. Initial notes were taken to capture recurring ideas, contrasts, and emotionally salient expressions.

2. Initial Coding

Each meaning unit was initially coded inductively at a semantic level, allowing codes to emerge directly from participants’ responses. Subsequently, a deductive approach was applied to organize these codes into broader theoretical categories informed by the study’s conceptual framework on language, identity, and communication. Coding was conducted at a semantic level, prioritizing participants’ explicit statements rather than latent interpretation. One meaning unit was assigned one primary code.

3. Development of Theoretical Categories

Following initial inductive coding, codes were grouped into four overarching theoretical categories using a deductive approach informed by the study’s conceptual framework. These categories remained stable throughout the analysis:

- Linguistic Identity. How language shapes, reinforces, or challenges personal and social identity.
- Emotional Impact of Language. Εmotional responses elicited by specific terms, expressions, or narratives.
- Speaker and Context. Τhe influence of who speaks (e.g., healthcare professionals, media, social environment) and in what context.
- Preferences and Recommendations. Participants’ preferred terminology and suggestions for improving cancer-related communication.

No additional categories were introduced during later stages of analysis, indicating theoretical saturation at the category level.

4. Refinement and Consolidation

Codes were reviewed for internal consistency and conceptual clarity. Similar codes were merged where appropriate, while preserving meaningful distinctions. A codebook was developed, including code definitions and examples, and was applied consistently across all participants.

#### 2.2.5. Reliability and Analytical Rigor

Several strategies were employed to enhance methodological rigor:

- Consistency of coding structure: the same coding framework and table structure were applied to all participants.
- Audit trail: coding decisions, category definitions, and revisions were documented throughout the process.
- Negative and deviant cases were retained and analyzed to ensure that dominant patterns did not overshadow divergent experiences.
- Data saturation was assessed iteratively; no new categories emerged after the inclusion of later participants.

#### 2.2.6. Data Organization and Quantification

Coded data were organized in structured tables, allowing for both qualitative interpretation and descriptive quantification. Frequency counts were calculated for each code, indicating the number of references, and the number of participants contributing to each code. These frequencies were used to identify dominant patterns without implying statistical generalization.

### 2.3. Ethical Considerations

The study was conducted in accordance with the Declaration of Helsinki and was approved by the Ethics Committee of the International Hellenic University (approval number ΔΦ 15/6438/10-04-2024). Participation was voluntary and anonymous. All participants received information about the study aims and procedures and provided informed consent prior to participation. No identifying information was included in the dataset. Responses were analyzed and reported in a way that preserved participants’ dignity and respected the sensitivity of lived cancer experiences.

## 3. Results

### 3.1. Sample Characteristics Phase I

The quantitative phase included a total of 146 participants with a confirmed diagnosis of cancer (Table 1). Of the total sample, 74.0% were women (*n* = 108) and 26.0% were men (*n* = 38). Participants’ age ranged from 22 to 82 years, with a mean age of 54.35 years (SD = 11.32). Valid age data were available for 145 participants. Participants were also asked to indicate how they would prefer to be described in relation to their illness. Responses revealed considerable variation in preferred terminology. The most frequently selected descriptors were “cancer patient” (25.3%) and “oncology patient” (24.7%), followed by “patient/person with cancer” (16.4%) and “patient/person with neoplastic disease” (13.0%). A smaller proportion of participants indicated that they preferred an alternative description (6.8%), while 13.7% reported indifference toward the terminology used. Regarding the emotional impact of illness-related labels, responses indicated that the term “patient” was generally perceived as less emotionally burdensome compared to “cancer patient.” Emotional responses to the term “patient” ranged from neutral to mildly negative (M = 3.74, SD = 1.12), whereas the term “cancer patient” elicited more negative emotional evaluations overall (M = 3.38, SD = 1.36). These findings are further examined in subsequent inferential analyses.

### 3.2. Sample Characteristics Phase II

The final sample consisted of 11 participants with lived experience of cancer. The majority of participants were women (*n* = 8). Participants’ ages ranged from 41 to 73 years, reflecting a broad adult age spectrum. All participants had completed tertiary education, with most holding a postgraduate degree, indicating a highly educated sample. Participants resided in diverse geographic settings across Greece, including urban areas, provincial regions, islands, and rural contexts, allowing for variation in social and healthcare environments. A range of cancer types was represented, including breast cancer, Hodgkin lymphoma, lung cancer, and other malignant neoplasms. Most participants reported a progressed or advanced stage of disease at the time of diagnosis, while a smaller number had been diagnosed at an earlier stage. Time since diagnosis varied substantially, from recent diagnoses to long-term survivorship exceeding eight years, enabling the inclusion of perspectives from different phases of the cancer trajectory. All participants had undergone medical treatment, including surgery, chemotherapy, radiotherapy, or combinations thereof. This heterogeneity in diagnosis, disease stage, and treatment experience provided a rich context for exploring how cancer-related language is perceived and interpreted across different clinical and temporal positions. A detailed overview of participants’ sociodemographic and clinical characteristics is provided in Table 2.

### 3.3. Results of Phase I

#### 3.3.1. Emotional Impact of Illness-Related Terminology

Descriptive statistics indicated that the two illness-related labels were not experienced equivalently by participants. As shown in Table 3, the term “patient” was associated with a higher mean score (M = 3.74, SD = 1.12), indicating a less negative emotional impact compared to the term “cancer patient” (M = 3.38, SD = 1.36).

To formally examine this difference, a Wilcoxon signed-rank test was conducted on paired observations. The analysis revealed a statistically significant difference between the two terms (Z = −4.38, *p* < 0.001), indicating that participants consistently evaluated the label “cancer patient” as more emotionally negative than the label “patient” (Table 4). The direction of ranks further showed that negative emotional evaluations were more frequently associated with the cancer-specific label.

#### 3.3.2. Predictors of Preferred Illness-Related Description

A multinomial logistic regression analysis was performed to investigate whether sociodemographic characteristics and emotional responses to terminology predicted participants’ preferred illness-related description. Preferred terminology served as the dependent variable, with “indifferent” used as the reference category. Age, gender, emotional impact of the term “patient,” and emotional impact of the term “cancer patient” were included as predictors. The overall model demonstrated a statistically significant improvement over the intercept-only model (likelihood ratio χ^2^ = 40.95, df = 20, *p* = 0.004). As summarized in Table 5, age and gender were not significantly associated with preferred terminology across any comparison category (all *p* > 0.05). Similarly, the emotional impact of the generic term “patient” did not emerge as a significant predictor. In contrast, the emotional impact of the term “cancer patient” was a significant predictor of terminology preference (χ^2^ = 22.64, df = 5, *p* < 0.001). Specifically, greater negative emotional evaluation of this label was associated with a reduced likelihood of preferring alternative illness-related descriptors, such as “oncology patient,” “patient/person with neoplastic disease,” or “other”, relative to reporting indifference. This finding suggests that increased emotional burden linked to cancer-specific labeling is associated with distancing from illness-related categorization more broadly.

#### 3.3.3. Summary of Quantitative Findings

Taken together, the results of Phase I indicate that reactions to cancer-related language are not shaped by basic sociodemographic factors such as age or gender. Instead, participants’ preferences appear to be primarily driven by the emotional meaning attached to specific terms, with the label “cancer patient” emerging as both more emotionally negative and more influential in shaping terminology preferences.

### 3.4. Results of Phase II

#### 3.4.1. Overview of the Content Analysis

The findings are organized into four analytical blocks reflecting the overarching theoretical categories derived from the content analysis. Each block synthesizes key patterns across participants’ narratives and illustrates how language related to cancer shapes identity, emotional experience, and communication practices (Table 6).

#### 3.4.2. Linguistic Identity

Labeling/Stigma vs. Person-First Language—Survivorship as Reframing—Rejection of Heroization/Victimization.

Participants consistently emphasized that cancer-related terminology plays a central role in shaping personal identity. Disease-centered labels were often experienced as stigmatizing, as they appeared to reduce individuals to their diagnosis and obscure other dimensions of the self. In contrast, person-first expressions (e.g., “a person who has had cancer”) were perceived as preserving individuality and acknowledging cancer as one aspect of a broader life narrative.

The concept of “survivor” emerged as a form of identity reframing, particularly among participants who had completed active treatment. Survivorship was described not as a permanent identity but as a way to signal continuity, resilience, and movement beyond illness. Importantly, participants distinguished between acknowledging survival and allowing cancer to define who they are. At the same time, participants rejected polarized representations that framed people with cancer either as heroes or as victims. Such narratives were seen as reductive and ethically problematic, as they impose moral meanings on health outcomes and fail to capture the complexity of lived experience. Instead, participants advocated for language that recognizes cancer as a serious condition without transforming it into a defining or moralizing identity.

#### 3.4.3. Emotional Impact of Language

Fear and Fatalistic Framing, Empowering Language, Neutrality and Indifference.

Language was described as having a direct emotional impact, capable of evoking fear, distress, reassurance, or neutrality. Terms and expressions associated with fatalism, such as euphemisms implying inevitability or death, were frequently linked to anxiety and emotional burden. Participants highlighted that such wording can reinforce perceptions of cancer as a “sentence” rather than a condition with variable trajectories. Conversely, language that conveyed support, care, and realistic acknowledgment of the situation was experienced as emotionally grounding and, in some cases, empowering. Expressions of presence, solidarity, and honest reassurance were valued more than exaggerated optimism or dramatic framing.

Notably, some participants reported a largely neutral or indifferent emotional response to cancer-related terminology. This stance was often associated with time since diagnosis, personal coping strategies, or familiarity with medical discourse. The presence of neutrality underscores that emotional responses to language are not uniform and may evolve over time.

#### 3.4.4. Speaker and Context

The Physician’s Role, Social Environment, Media and Public Discourse.

Participants consistently underscored that the impact of language depends heavily on who speaks and in what context. Physicians were attributed particular authority and responsibility; from healthcare professionals, participants expected clarity, accuracy, and empathy. Language used by clinicians was perceived as especially influential, shaping not only understanding but also trust and emotional security. The social environment played a more ambivalent role. Family members and friends were sometimes described as supportive, yet their language could also feel intrusive, minimizing, or emotionally demanding. Participants often negotiated or filtered social interactions to protect themselves from distressing or unhelpful communication. Media and public discourse were frequently criticized for oversimplification and sensationalism. Participants described frustration with portrayals that oscillate between extremes, depicting individuals with cancer as either heroes or victims, and with the prevalence of overinformation and misinformation, particularly on social media platforms. Such representations were viewed as contributing to fear, stigma, and emotional overload.

#### 3.4.5. Preferences and Recommendations

Naming Cancer Directly, Education and Media Filtering. Holistic and Peer-Oriented Care.

Across narratives, participants expressed clear preferences regarding how cancer should be discussed. Many emphasized the importance of naming cancer directly, rejecting euphemisms that obscure reality and reinforce fear. Using clear and honest terminology was seen as a prerequisite for normalization and destigmatization. Participants also called for broader education, both of healthcare professionals and the general public, focused on communication skills, emotional awareness, and responsible use of language. Particular emphasis was placed on the need to filter and regulate information circulated through social media to reduce misinformation and emotional harm. Finally, participants highlighted the value of holistic approaches to care, including psychological, social, and emotional support alongside medical treatment. Peer support and spaces for shared experience were identified as especially meaningful, offering forms of understanding and validation that extend beyond formal healthcare encounters.

#### 3.4.6. Frequency Patterns Across Codes

Analysis of code frequencies highlights several dominant and cross-cutting patterns in participants’ accounts. The role of the speaker and communicative context emerged as the most pervasive dimension of meaning-making. The code “Role of speaker and context” was identified in the narratives of the majority of participants (*n* = 9) and generated the highest number of references (15), underscoring that the perceived impact of cancer-related language depends strongly on who speaks (e.g., healthcare professionals, family members, media) and under what circumstances (Table 7).

Within the Linguistic Identity category, issues of stigma and identity negotiation were particularly prominent. Codes related to stigma or devalued identity (13 references, *n* = 8) and the rejection of stigmatizing terms (12 references, *n* = 7) indicate widespread resistance to language that defines individuals primarily through their illness. Participants frequently described efforts to distance their personal identity from the disease, as reflected in the recurrent presence of loss or separation of identity (10 references, *n* = 6) and identity reframing through survivorship (8 references, *n* = 5). The parallel appearance of normalization of the disease (8 references, *n* = 5) further suggests a collective move away from exceptionalizing or moralizing cancer.

Regarding the Emotional Impact of Language, both negative and positive emotional responses were evident. Negative emotional reactions (14 references, *n* = 7) and fear or fatalistic framing (8 references, *n* = 6) coexisted with expressions of empowerment through language (11 references, *n* = 7) and neutral or non-negative emotional responses (9 references, *n* = 6). This distribution illustrates that language can simultaneously function as a source of distress, reassurance, or emotional neutrality, depending on individual circumstances and temporal distance from diagnosis.

Finally, within Preferences and Recommendations, participants articulated clear expectations for how cancer should be communicated. The most frequently endorsed needs were empathic communication (14 references, *n* = 8) and destigmatizing, realistic language (13 references, *n* = 8). Substantial emphasis was also placed on person-first or neutral terminology (11 references, *n* = 7), clear and trustworthy information (10 references, *n* = 7), and education of both professionals and the wider public (9 references, *n* = 6). Together, these findings point to a strong collective demand for communication practices that combine honesty, sensitivity, and respect.

## 4. Discussion

The aim of the present study was to explore how cancer-related language is perceived and experienced by individuals with lived experience of cancer, and to examine the ways in which terminology shapes identity, emotional responses, and communication practices. The findings highlight that language surrounding cancer is far from neutral; rather, it functions as a powerful social and emotional construct that influences how individuals understand themselves, how they are positioned by others, and how communication unfolds across clinical, social, and public contexts. The quantitative results demonstrated clear patterns in terminology preferences and emotional associations, while the qualitative findings provided in-depth insight into the meanings, interpretations, and lived experiences underlying these patterns. Taken together, the complementary use of quantitative and qualitative methods allowed for a nuanced understanding of cancer-related language, revealing both its measurable effects and its subjective significance.

### 4.1. Language as a Determinant of Identity and Social Meaning

Findings from Phase II highlight language as a central mechanism through which identity, stigma, and social meaning are negotiated in the cancer experience. Participants’ accounts demonstrate that diagnostic labels and illness-centered terminology operate not merely as descriptive tools, but as socially embedded practices that actively shape self-perception and interpersonal relations. Across qualitative studies included in the dataset, disease-first labels were experienced as stigmatizing and reductive, reinforcing an identity narrowly defined by illness and marginalizing other dimensions of personhood [25,26,27].

Participants described illness-centered labels as markers of social stigma, associated with shame, guilt, and constrained disclosure, particularly in cultural contexts where cancer is linked to fatalism or moral interpretations [10,26]. These findings align with broader qualitative evidence demonstrating that stigma is enacted not only through overt discrimination but also through everyday language that collapses the person into the diagnosis. Labeling practices contributed to identity disruption or “biographical fracture,” as individuals struggled to reconcile pre-illness and illness-related identities [28,29,30].

In contrast, person-first language emerged as a deliberate linguistic strategy through which individuals sought to preserve continuity of self and resist identity foreclosure. Participants emphasized that formulations placing the person before the disease enabled them to assert agency, maintain social roles, and re-establish a sense of personal integrity beyond biomedical categorization [31,32]. Evidence from studies on patient–clinician communication and values elicitation further supports the role of person-centered language in reinforcing autonomy and mitigating psychosocial distress. Respectful linguistic framing was associated with enhanced trust, emotional validation, and a stronger sense of being recognized as a whole person rather than a clinical case [33].

The notion of survivorship was articulated by participants not as a permanent or essentialized identity, but as a flexible narrative resource for meaning-making. Survivorship functioned as a way of reinterpreting the cancer experience without requiring continued identification with illness or adherence to socially valorized recovery narratives [3,4]. This framing challenges dominant survivorship discourses that implicitly assume linear recovery and stable post-treatment identities. Instead, survivorship was described as provisional, context-dependent, and intertwined with ongoing uncertainty, bodily vulnerability, and emotional fluctuation, findings consistent with narrative accounts of adaptation and reconstruction rather than closure [30,31].

Participants explicitly rejected dichotomous framings that positioned individuals with cancer either as heroic fighters or passive victims. These narratives were perceived as moralizing and simplificatory, imposing normative expectations regarding optimism, resilience, or strength, while delegitimizing fear, ambivalence, and suffering [28,34]. Such binary constructions intensified emotional labor and constrained authentic self-expression, particularly when individuals felt compelled to perform socially acceptable illness identities. The rejection of these framings reflects resistance to moral hierarchies embedded in dominant cancer discourses and underscores the need for narrative spaces that accommodate complexity, contradiction, and vulnerability [29,31]. At the same time, it is important to acknowledge that such terminology is not inherently negative and may reflect positive intentions. Terms such as “fighter” or “survivor” are often used to convey strength, resilience, and active engagement with illness, drawing on culturally embedded metaphors of struggle and endurance. For some individuals, these expressions may serve as empowering narratives that support coping, reinforce agency, and provide a sense of meaning or identity during the cancer trajectory. From this perspective, such language may function as a psychological resource rather than a constraint.

### 4.2. Emotional Impact of Cancer Terminology

The findings of this study indicate that cancer-related terms are not emotionally neutral markers; rather, they act as affective triggers whose impact varies substantially across individuals and contexts [35].

Fear and anxiety emerged as prominent reactions, reflecting broader evidence that cancer-related language amplifies threat perceptions and anticipatory distress [36]. Similarly, population-level sentiment work shows that negative emotional clusters around cancer topics commonly include fear and sadness, and are linked to stigma and avoidance dynamics [9]. In diagnostic disclosure settings, intense emotional distress (e.g., anxiety, sadness, despair, sleep disturbance) is described as immediate and identity-relevant, reinforcing social withdrawal and isolation [37].

At the same time, Phase II data also captured empowerment not as a universal response to cancer language, but as a conditional outcome when terminology is paired with autonomy, privacy, and control. For example, self-directed screening approaches have been described as more acceptable and emotionally manageable because they enhance agency and reduce stigma exposure [38]. In clinical communication more broadly, scholars argue that terminology carries ethical and psychosocial weight, words can evoke fear and stigma, or conversely provide reassurance and clarity when used precisely and empathetically [39].

A third response pattern, neutrality, was also evident. Importantly, neutrality should not be misread as emotional absence, but rather as a potential indicator of adaptation, desensitization, or successful meaning integration over time. Qualitative work on illness temporality and identity renegotiation shows that emotional and interpretive responses evolve across “cancer time,” as individuals re-author experience, adjust biography, and re-stabilize self-concepts [40,41]. Evidence from prostatectomy narratives likewise illustrates how neutrality can coexist with satisfaction and “relief,” reflecting an accommodation process rather than a lack of affect [42].

The negative evaluation of the label “cancer patient” suggests that illness-related terminology is affectively loaded and may activate culturally embedded meanings of threat, fatality, and stigma [43]. When such scripts are activated, fear becomes not simply an individual emotion but a socially patterned response linked to stigma and fatalistic beliefs that shape avoidance, disengagement, and distress [36,44]. This quantitative signal is strengthened by qualitative accounts in the dataset where fear is narrated as (a) fear of outcomes and recurrence, (b) fear of social exposure and stigma, and (c) fear shaped by inadequate information or emotionally insufficient communication [45]. In other words, the label’s negative emotional valence can be understood as a convergence point between social meaning (culturally shared connotations) and psychological experience (anxiety, uncertainty), producing the statistically detectable effect observed in the quantitative strand. Notably, the impact of terminology is not restricted to feelings—it may also influence decisions and care trajectories. For instance, it has been argued that the category “carcinoma,” when applied to clinically indolent conditions, can drive patient acceptance of aggressive interventions through fear triggered by “cancer terminology” [46]. This supports the interpretation that negatively charged labels may shape both affect and behavior by amplifying perceived threat.

Phase II narratives suggest that neutrality may emerge through temporal distance from diagnosis and personal coping strategies that reframe cancer as one element of life rather than a totalizing identity. Longitudinal narrative evidence shows that over time individuals renegotiate “self as embodied,” “self in relationships,” and “self in place,” which can soften initial affective spikes and allow more neutral linguistic appraisals [41]. In family contexts, coping practices (e.g., acceptance, religious coping, communication with providers) similarly appear to re-regulate fear and distress, especially when illness becomes integrated into everyday routines rather than remaining an acute rupture [45,47]. Quantitative work on distress trajectories also supports the plausibility of “neutrality through adaptation,” as emotional distress can reduce when supportive interventions promote reappraisal, self-acceptance, and emotional regulation [48]. Likewise, distress screening studies demonstrate that emotional states fluctuate and can persist beyond acute treatment, suggesting that neutrality is not guaranteed, but may reflect successful adjustment for some subgroups while others remain vulnerable [49].

### 4.3. The Role of the Speaker and the Communicative Context

One of the strongest findings of the present study concerns the decisive role of the speaker and the communicative context in shaping the emotional and interpretive impact of cancer-related language. Phase II results indicate that identical terminology may be experienced as reassuring, threatening, or emotionally neutral depending on who uses it, where, and within which relational framework. This finding aligns with empirical evidence demonstrating that language in oncology is inherently context-dependent and relationally mediated rather than semantically fixed.

Health professionals occupy a dual communicative position: they function simultaneously as carriers of epistemic authority and as potential sources of emotional safety or insecurity. Research on doctor–patient communication consistently shows that patients attribute heightened weight and legitimacy to clinicians’ words, particularly those of physicians, whose language is interpreted as consequential for prognosis, decision-making, and existential meaning [21,50]. High-quality clinical communication characterized by clarity, accuracy, and empathic attunement has been shown to buffer distress and foster trust, even under conditions of heightened uncertainty or fear [20]. Conversely, imprecise, emotionally detached, or jargon-heavy language may intensify anxiety and hopelessness, reinforcing feelings of vulnerability and loss of control. Importantly, discrepancies between physician-reported and patient-reported outcomes further highlight that communication is not merely informational but interpretive, with patients assigning emotional meaning that may diverge from clinical intent [51].

Participants expressed distinct expectations depending on the speaker, particularly emphasizing accuracy and empathy in physician communication [21,52,53]. By contrast, communication originating from the social environment was frequently described as ambivalent. While such interactions can offer emotional support, they may also reproduce fear, misinformation, or moral pressure, particularly when influenced by cultural taboos, fatalistic beliefs, or avoidance of open discussion [22,54]. This ambivalence underscores that non-professional communicators lack the institutional authority of clinicians but nonetheless exert substantial emotional influence.

Media representations of cancer constitute a distinct communicative context in which language is routinely dramatized, oversimplified, and embedded in stereotypical narratives. Empirical analyses show that cancer is frequently framed as crisis, battle, or moral test, employing emotionally charged language that amplifies fear while marginalizing uncertainty and everyday illness trajectories [34,55]. Such oversimplification reduces complex experiences to linear scripts of survival or failure, shaping public prototypes of cancer and influencing symptom appraisal and help-seeking behavior [56,57]. Moreover, media discourse often reproduces stigmatizing stereotypes by implicitly linking cancer to personal responsibility or moral judgment, particularly for certain diagnoses [24,58].

It is also important to consider that the predominance of female participants in the sample may have shaped these findings, as gendered differences in emotional processing and communication preferences could influence how cancer-related language is experienced and interpreted.

### 4.4. Theoretical and Practical Implications

The findings of this study make a substantive contribution to psychosocial oncology and the study of medical communication by demonstrating that language is not a neutral conduit of information but a context-sensitive psychosocial intervention that actively shapes identity, coping, and emotional regulation. By foregrounding the concept of context-dependent language impact, the study extends narrative and identity-based approaches in psycho-oncology, showing how linguistic framing intersects with embodiment, survivorship trajectories, and stigma management [12,14]. At the level of medical communication, the results underscore the relational and ethical dimensions of terminology use, reinforcing evidence that imprecise, moralized, or standardized labels may undermine agency and trust, whereas person-attuned and narratively flexible language can foster engagement and psychosocial safety [13,59]. Practically, these insights have direct implications for clinical practice, supporting the need for communication training that emphasizes contextual sensitivity, patient-preferred terminology, and awareness of the emotional load carried by diagnostic labels. Beyond the clinic, the findings call for greater reflexivity in public discourse and media representations, where dramatized and stereotypical language may amplify fear and stigma. Finally, at the level of health policy, the study supports integrating psycho-oncological principles and communication standards into cancer care pathways and public health strategies, positioning language as a modifiable determinant of patient experience and equity in cancer care [60]. Overall, these findings suggest that small shifts in language use may have meaningful effects on patient experience, highlighting communication as a key site for intervention in cancer care.

### 4.5. Limitations and Directions for Future Research

Several limitations of the present study should be acknowledged. First, the small qualitative sample size restricts the transferability of the findings, as the results primarily reflect in-depth experiences rather than population-level patterns. Second, the high educational level of participants may have shaped both linguistic awareness and reflexivity, potentially amplifying sensitivity to terminology and communication practices compared to more socioeconomically diverse populations. These characteristics suggest caution in generalizing the findings to groups with lower health literacy or limited access to communicative resources. In addition, the sample was characterized by a gender imbalance, with a predominance of female participants, which may have influenced the findings. Gender differences in communication styles, emotional expression, and illness experience are well documented, and it is possible that the perspectives captured in this study more strongly reflect female experiences of cancer-related language. In particular, preferences for softer, relational, or neutral expressions may be more prominently represented, whereas more militaristic or “fighter”-oriented metaphors may be perceived differently across genders. Future research would benefit from longitudinal designs to examine how the emotional and identity-related impact of cancer language evolves across the illness trajectory, from diagnosis to long-term survivorship. Cross-cultural comparative studies are also warranted, given the culturally embedded meanings of cancer terminology and stigma. Finally, extending inquiry to healthcare professionals, exploring their linguistic choices, communicative constraints, and perceptions of emotional impact, would offer a more comprehensive understanding of cancer communication as a relational and institutional practice, strengthening the integration of psychosocial insights into clinical and policy contexts.

## 5. Conclusions

This study demonstrates that cancer-related language is not merely descriptive but actively constructs identity, shapes emotional experience, and influences communication dynamics across clinical, social, and media contexts. By integrating quantitative and qualitative approaches, it reveals that terminology such as “cancer patient” carries stigmatizing and emotionally charged connotations, while person-first and context-sensitive language fosters agency, emotional safety, and identity continuity. The role of the speaker emerges as pivotal, with participants underscoring the ethical and emotional weight of clinicians’ words. Furthermore, euphemistic or dramatized media portrayals were shown to amplify fear and moralize illness narratives, reinforcing the urgency for linguistic precision, empathy, and education in both medical and public spheres. Ultimately, language is not a neutral tool in cancer care, it is a modifiable determinant of dignity, trust, and psychological well-being.

## Figures and Tables

**Table 1:** Sociodemographic and descriptive characteristics of the Phase I sample (N = 146).

Variable	*n*	%/M (SD)
Gender		
Women	108	74.0
Men	38	26.0
Age (years)	145	54.35 (11.32)
Minimum–Maximum		22–82
Preferred illness-related description		
Patient/person with cancer	24	16.4
Oncology patient	36	24.7
Cancer patient	37	25.3
Patient/person with neoplastic disease	19	13.0
Other	10	6.8
Indifferent	20	13.7

Note. Percentages are calculated based on valid responses. Minor variations in sample size across variables are due to missing data.

**Table 2:** Sociodemographic and clinical characteristics of qualitative participants (Phase II, N = 11).

Participant	Gender	Age Range	Cancer Type	Disease Stage at Diagnosis	Time Since Diagnosis
P01	Female	40–49	Breast cancer	Early stage	1–2 years
P02	Female	50–59	Breast cancer	Advanced stage	3–5 years
P03	Male	60–69	Lung cancer	Advanced stage	1–2 years
P04	Female	50–59	Hodgkin lymphoma	Early stage	>5 years
P05	Female	40–49	Breast cancer	Advanced stage	1–2 years
P06	Male	60–69	Lung cancer	Advanced stage	3–5 years
P07	Female	50–59	Breast cancer	Early stage	1–2 years
P08	Female	60–69	Breast cancer	Early stage	>5 years
P09	Female	40–49	Other	Advanced stage	3–5 years
P10	Male	60–69	Other	Advanced stage	>5 years
P11	Female	50–59	Breast cancer	Early stage	>5 years

**Table 3:** Descriptive statistics for emotional impact of illness-related labels (Phase I).

Label	N	Mean	SD	Minimum	Maximum
Patient	140	3.74	1.12	1	5
Cancer patient	137	3.38	1.36	1	5

Note. Higher scores indicate less negative emotional impact. Minor variations in N are due to missing data.

**Table 4:** Wilcoxon signed-rank test comparing emotional impact of the labels “patient” and “cancer patient”.

Comparison	N (Pairs)	Z	*p*
Cancer patient—Patient	134	−4.38	<0.001

Note. Negative Z value indicates that the term “cancer patient” was evaluated as more emotionally negative than “patient”.

**Table 5:** Multinomial logistic regression predicting preferred illness-related description (reference category: Indifferent).

Predictor	χ^2^	df	*p*
Age	2.93	5	0.711
Gender	2.98	5	0.704
Emotional impact of “patient”	1.83	5	0.872
Emotional impact of “cancer patient”	22.64	5	<0.001

Note. Reported values are likelihood ratio tests. Only the emotional impact of the term “cancer patient” significantly predicted preferred illness-related description.

**Table 6:** Illustrative quotations by theoretical category.

Theoretical Category	Subtheme	Illustrative Quotation	Participant
Linguistic Identity	Labeling and stigma vs. person-first language	“It feels as if my identity as a person is taken away and suddenly replaced by an identity defined by the illness.”	P09
		“I don’t want cancer to be my identity. It’s something I went through, not who I am.”	P11
	Survivorship as reframing	“I correct people by saying I’m no longer a patient. I belong to the survivors now.”	P08
	Rejection of hero/victim narratives	“I don’t want to be presented as someone to pity, but not as a hero either. I’m just a person who went through a serious illness.”	P11
Emotional Impact of Language	Fear and fatalistic framing	“The phrase ‘terminal illness’ really bothers me. It makes cancer sound like a death sentence.”	P06
		“malignant disease” carries fear and fatalism, as if cancer is unspeakable and inevitably deadly.”	P09
	Empowering and supportive language	“Hearing ‘I’m here for whatever you need’ makes a huge difference. It feels supportive without exaggeration.”	P08
	Neutrality and emotional distance	“Honestly, it doesn’t really affect me. I’ve become quite neutral toward these terms.”	P10
Speaker and Context	Authority of healthcare professionals	“What my doctor says carries weight. I need clear and honest information from them.”	P07
		“From doctors I expect sensitivity and respect: every word matters more than they might think.”	P09
	Social environment	“People mean well, but sometimes their words are stressful and make me feel more like a patient.”	P05
	Media and public discourse	“The media show either heroes or victims. Real life after cancer has many shades.”	P09
Preferences and Recommendations	Naming cancer directly	“We should say the word ‘cancer’ openly, instead of hiding behind euphemisms.”	P11
	Education and communication training	“Professionals need to be educated in how to communicate with empathy, not just medically.”	P06
	Holistic and peer-oriented support	“I would trust people who have lived it. We need spaces where patients can talk to each other.”	P04

Note. Quotations were selected to represent dominant patterns within each theoretical category while preserving variation in experiences and perspectives. Participant identifiers (P01–P11) ensure anonymity.

**Table 7:** Frequency distribution of codes by theoretical category.

Code	Theoretical Category	Number of References	Participants (*n* = 11)
Negative emotional response	Emotional Impact of Language	14	7
Neutral/non-negative emotional response	Emotional Impact of Language	9	6
Empowerment through language	Emotional Impact of Language	11	7
Fear/fatalistic framing	Emotional Impact of Language	8	6
Stigma/devalued identity	Linguistic Identity	13	8
Rejection of stigmatizing terms	Linguistic Identity	12	7
Loss or separation of identity (person vs. disease)	Linguistic Identity	10	6
Reframing identity (survivor/survivorship)	Linguistic Identity	8	5
Normalization of the disease (no heroization/pity)	Linguistic Identity	8	5
Role of speaker and context	Speaker and Context	15	9
Influence of media/public discourse	Speaker and Context	10	7
Overinformation/misinformation	Speaker and Context	7	5
Person-first approach/neutral terminology	Preferences and Recommendations	11	7
Need for empathic communication	Preferences and Recommendations	14	8
Destigmatizing/realistic language	Preferences and Recommendations	13	8
Need for education (society/professionals)	Preferences and Recommendations	9	6
Need for clear and trustworthy information	Preferences and Recommendations	10	7

Note. Frequencies are reported descriptively to indicate the distribution and prominence of themes within the sample and do not imply statistical comparison.

## Data Availability

The data presented in this study are available on reasonable request from the corresponding author due to ethical considerations and privacy restrictions.

## Supplementary Materials

The following supporting information can be downloaded at: https://doi.org/10.5281/zenodo.19512148 (accessed on 3 December 2025).

## Public Involvement Statement

Public involvement was integral to multiple stages of this research, consistent with the GRIPP2 (Guidance for Reporting Involvement of Patients and the Public) framework. The study was conducted in collaboration with the Cancer Patient Guidance Center (KAPA3), a non-profit organization supporting individuals living with cancer. Patient representatives and advocates with lived experience were actively engaged as research partners rather than solely as participants.

## Guidelines and Standards Statement

This manuscript was drafted in accordance with established reporting guidelines for mixed-methods research. The quantitative component was reported following the STROBE (Strengthening the Reporting of Observational Studies in Epidemiology) guidelines for cross-sectional studies, while the qualitative component was informed by the COREQ (Consolidated Criteria for Reporting Qualitative Research) checklist.
